# Supplementary material for: Modeling glioblastoma heterogeneity as a dynamic network of cell states
Source: Mol Syst Biol. 2021 Sep 16;17(9):e10105. doi: 10.15252/msb.202010105 (PMC8444284; doi:10.15252/msb.202010105)
Supplement: Supplementary file 6 — Source Data for Figure 5 [file MSB-17-e10105-s004.zip › Figure5A_sourcedata/GSEA_3017/nosigdb_stateA.GseaPreranked.1622795026485/GANDAL_SCHIZOPHRENIA_UP_PMID29439242.html]

Details for gene set GANDAL\_SCHIZOPHRENIA\_UP\_PMID29439242[GSEA]

|  || Dataset | state53017 |
| Phenotype | NoPhenotypeAvailable |
| Upregulated in class | na\_neg |
| GeneSet | GANDAL\_SCHIZOPHRENIA\_UP\_PMID29439242 |
| Enrichment Score (ES) | -0.5423331 |
| Normalized Enrichment Score (NES) | -2.5140493 |
| Nominal p-value | 0.0 |
| FDR q-value | 1.0676692E-4 |
| FWER p-Value | 0.002 |
Table: GSEA Results Summary

  

Fig 1: Enrichment plot: GANDAL\_SCHIZOPHRENIA\_UP\_PMID29439242      
 Profile of the Running ES Score & Positions of GeneSet Members on the Rank Ordered List

  

| PROBE | GENE SYMBOL | GENE\_TITLE | RANK IN GENE LIST | RANK METRIC SCORE | RUNNING ES | CORE ENRICHMENT || 1 | HSPB1 |  |  | 312 | 0.322 | -0.3075 | No |
| 2 | FKBP5 |  |  | 368 | 0.304 | -0.3467 | No |
| 3 | HMGB2 |  |  | 424 | 0.288 | -0.3869 | No |
| 4 | MT2A |  |  | 473 | 0.275 | -0.4205 | No |
| 5 | PSAT1 |  |  | 491 | 0.271 | -0.4219 | No |
| 6 | VEGFA |  |  | 546 | 0.261 | -0.4627 | No |
| 7 | SOX9 |  |  | 577 | 0.254 | -0.4787 | No |
| 8 | AGT |  |  | 587 | 0.252 | -0.4728 | No |
| 9 | CD99 |  |  | 604 | -0.252 | -0.4743 | No |
| 10 | PLIN3 |  |  | 619 | -0.259 | -0.4732 | No |
| 11 | ARHGEF26 |  |  | 628 | -0.262 | -0.4657 | No |
| 12 | GNG12 |  |  | 640 | -0.265 | -0.4611 | No |
| 13 | ABCA1 |  |  | 647 | -0.268 | -0.4511 | No |
| 14 | ELOVL2 |  |  | 735 | -0.329 | -0.5224 | Yes |
| 15 | PON2 |  |  | 743 | -0.338 | -0.5092 | Yes |
| 16 | SLC4A4 |  |  | 763 | -0.355 | -0.5076 | Yes |
| 17 | ITGB8 |  |  | 774 | -0.363 | -0.4960 | Yes |
| 18 | MLC1 |  |  | 783 | -0.369 | -0.4820 | Yes |
| 19 | GJA1 |  |  | 823 | -0.405 | -0.4983 | Yes |
| 20 | PLOD2 |  |  | 838 | -0.431 | -0.4868 | Yes |
| 21 | TGFB2 |  |  | 840 | -0.432 | -0.4616 | Yes |
| 22 | SLC1A2 |  |  | 860 | -0.450 | -0.4542 | Yes |
| 23 | IFITM2 |  |  | 864 | -0.460 | -0.4295 | Yes |
| 24 | F3 |  |  | 888 | -0.526 | -0.4217 | Yes |
| 25 | ATP1B2 |  |  | 904 | -0.558 | -0.4035 | Yes |
| 26 | SDC2 |  |  | 908 | -0.568 | -0.3722 | Yes |
| 27 | SPP1 |  |  | 927 | -0.630 | -0.3528 | Yes |
| 28 | CEBPD |  |  | 939 | -0.690 | -0.3224 | Yes |
| 29 | IFITM3 |  |  | 954 | -0.830 | -0.2867 | Yes |
| 30 | CD44 |  |  | 957 | -0.851 | -0.2371 | Yes |
| 31 | CNN3 |  |  | 961 | -0.883 | -0.1867 | Yes |
| 32 | LGALS3 |  |  | 970 | -0.989 | -0.1351 | Yes |
| 33 | SLC1A3 |  |  | 973 | -1.097 | -0.0706 | Yes |
| 34 | LPL |  |  | 980 | -1.387 | 0.0073 | Yes |
Table: GSEA details [plain text format]

  

Fig 2: GANDAL\_SCHIZOPHRENIA\_UP\_PMID29439242: Random ES distribution      
 Gene set null distribution of ES for **GANDAL\_SCHIZOPHRENIA\_UP\_PMID29439242**
